# Future Perspective: Harnessing the Power of Artificial Intelligence in the Generation of New Peptide Drugs

**DOI:** 10.3390/biom14101303

**Published:** 2024-10-15

**Authors:** Nour Nissan, Mitchell C. Allen, David Sabatino, Kyle K. Biggar

**Affiliations:** 1 Institute of Biochemistry, Departments of Biology & Chemistry, Carleton University, Ottawa, ON K1S 5B6, Canadadavid.sabatino@carleton.ca (D.S.); 2 NuvoBio Corporation, Ottawa, ON K1S 5B6, Canada

**Keywords:** artificial intelligence, drug discovery, peptide drugs, computational tools, peptide design, screening

## Abstract

The expansive field of drug discovery is continually seeking innovative approaches to identify and develop novel peptide-based therapeutics. With the advent of artificial intelligence (AI), there has been a transformative shift in the generation of new peptide drugs. AI offers a range of computational tools and algorithms that enables researchers to accelerate the therapeutic peptide pipeline. This review explores the current landscape of AI applications in peptide drug discovery, highlighting its potential, challenges, and ethical considerations. Additionally, it presents case studies and future prospectives that demonstrate the impact of AI on the generation of new peptide drugs.

## 1. Introduction

**Figure 1:**
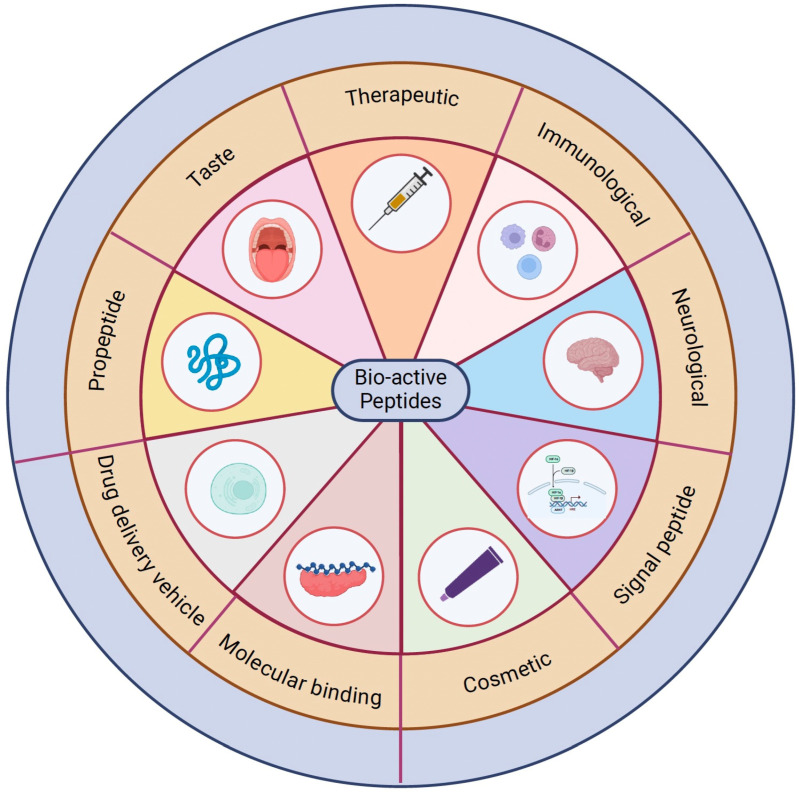
Select classes of bio-active peptides.

The rapidly emerging field of peptide-based drug discovery is filled with complex challenges and rewarding opportunities, aiming to identify and develop therapeutics that can effectively combat various diseases and improve patient outcomes. Peptide medications play a crucial role in effectively combating various diseases and enhancing patient outcomes. Currently, over 100 peptide drugs are available, particularly excelling in the area of glucose-lowering treatments with peptide such as GLP-1 analogs (ex. Liraglutide) [1,2]. Traditional methods of peptide drug discovery have relied on empirical approaches [3,4], which are often time-consuming, resource-intensive, and yield limited success rates. However, the rise of artificial intelligence (AI) has transformed drug discovery, creating exciting new opportunities for developing novel peptide drugs (Figure 1) [5]. AI is increasingly adopted in medicine, ranging from complex, high-cost applications to more accessible tools like large language models (LLMs). One notable example is ChatGPT, a leading LLM currently being explored for its potential applications in internal medicine and undergoing extensive study [6]. There have been recent advances in academic and industrial research partnerships towards the application of AI-based drug discovery platforms for peptide-based therapeutics [7]. Providing several key advantages over traditional small molecules, peptide-based drugs have gained significant attention in recent years due to their unique properties, such as high specificity and affinity, low toxicity, and potential for use against a wide range of disease targets [8]. Briefly, peptides are short chains of amino acids, typically consisting of fewer than 50 residues, and can modulate biological processes by interacting with specific receptors or proteins. As such, they are now recognized to hold immense therapeutic potential in diverse areas, including oncology and infectious diseases, immunology, neurology, and metabolic disorders [9,10,11,12,13].

AI encompasses a wide range of platforms, including machine learning, deep learning, natural language processing, and data mining, which enable the analysis and interpretation of vast amounts of biological and chemical data [14]. By leveraging these computational tools, researchers can expedite the peptide design, screening, and optimization processes, ultimately leading to the development of more effective peptide-based therapeutics [15]. The integration of AI techniques and computational tools in peptide drug discovery has emerged as a promising approach to overcome the limitations of traditional biochemical methods. Thus, the use of AI in the generation of new peptide drugs offers several key advantages. These include harnessing the predictive computational power of AI-generated algorithms for the in-silico discovery of new peptide ligands against therapeutic targets. Furthermore, AI algorithms can process and analyze large datasets, including genomic, proteomic, and structural data, to identify novel peptide sequences with desired properties [5]. The collection of bioinformatic data thus provides a conceptual framework that drives the collection of high-throughput data, and data mining for drug discovery [16]. This accelerates the discovery process and enables researchers to explore a broad chemical space. AI-driven predictive models can also provide valuable insights into peptide–protein interactions [17,18], aiding in the rational design of peptides with enhanced affinity and specificity for their targets. Moreover, AI algorithms can aid in the prediction of peptide bioactivity and toxicity [19], enabling early identification and prioritization of lead candidates.

Despite its immense potential, the implementation of AI in peptide drug discovery also poses challenges and considerations. AI models rely heavily on the quality and diversity of available training data [20], and the integration of multiple data types is crucial to capture the complexity of peptide–drug interactions. Therefore, the accuracy in the predictive power of AI-driven processes is often challenged by the complexity of the biological system. This review aims to explore the current landscape of AI applications in the generation of new peptide drugs. The review delves into various aspects of AI-driven peptide design, screening, and optimization, highlighting several case studies and notable discoveries. Furthermore, ethical and regulatory considerations associated with AI in peptide-based drug discovery and frameworks of prospectives and challenges in the field are outlined. By examining the transformative potential of AI in the generation of new peptide drugs, this narrative review aims to provide perspective insights into the future of drug discovery and its impact on patient care and therapeutics.

## 2. Traditional Approaches vs. AI in Peptide Drug Discovery

The traditional biochemical and medicinal chemistry approaches to peptide drug discovery have relied on experimental and empirical methods, which often entail a significant investment of time, resources, and expertise [21]. These approaches typically involve laborious trial-and-error processes, peptide library screenings, and, possibly, modifications based on limited structural information of varying experimental resolution and, as a result, confidence. While they have yielded notable successes, they are associated with several limitations that hinder efficiency and success rates in the preclinical-to-clinical translation of peptide drugs [8].

One of the primary challenges in traditional peptide drug discovery is the vast chemical space that needs to be explored [22]. The number of possible peptide sequences is exponentially large, making it impractical to test all potential candidates experimentally. This limitation restricts the ability to fully exploit the therapeutic potential of peptides and hinders the discovery of novel peptide scaffolds with desirable properties. Furthermore, rational design approaches rely on intuition and empirical knowledge, which can be subjective and biased. Designing peptides that exhibit high affinity and specificity for their intended targets requires a deep understanding of complex protein interactions, which may not always be feasible through a single “snapshot” of a structural-based peptide–protein interaction from a crystal structure, for example [23].

The traditional rational design and laboratory screening approach of novel peptide development is widely considered to be both costly and laborious in nature. As a result, many peptide therapeutics developed to date consist of endogenous peptides, or analogues of those peptides with known therapeutic properties (e.g., peptide hormones). Prior to the advent of synthetic methodologies, peptide drugs were developed solely through the discovery and isolation of functional peptides from animal tissue. These early therapeutic peptides mostly consisted of hormones isolated from tissues involved in specific metabolic pathways. For example, the peptide hormone insulin was isolated from pancreatic tissue following the discovery that removal of the pancreas led to diabetes in animal models [24]. Decades later, advances in solid-phase peptide synthesis enabled the development of a diverse array of peptides with an enormous variety of potential applications. Even so, the design of therapeutic peptides remained largely confined to analogues of native peptides [25,26]. Despite the modern advances in peptide design, synthesis, and screening approaches, the development of peptide analogues often remains favoured over completely novel sequences [27,28], largely as a consequence of persisting cost discrepancies between the two approaches. The continued development and implementation of AI in peptide design could vastly improve the feasibility of screening large libraries of peptides. AI enables the exploration of new chemical space, in silico, leading to the discovery of novel peptide ligands against complex biological targets, thereby significantly accelerating the peptide drug discovery process.

Challenges associated with traditional peptide screening and iterative design approaches are limited to the optimization of a peptide’s functional properties. There are a number of pharmacologically relevant properties which require extensive consideration and screening [29]. Many peptide analogues are modified exclusively to improve properties such as bioavailability and solubility relative to their native counterparts, rather than modifications that directly improve therapeutic activity [30,31]. Additionally, chemical modifications to the peptide backbone or amino acid substitutions are often necessary to improve therapeutic efficacy, and for protection against enzymatic degradation in plasma or the digestive system. For example, C-type natriuretic peptide is used to treat achondroplasia by promoting bone growth, but is highly susceptible to enzymatic degradation [32]. The synthetic analogue vosoritide addresses this therapeutic limitation through the addition of proline and glycine residues at the N-terminus, which significantly improves the stability of the peptide [33]. Resistance to peptidase degradation can also be achieved through the addition of a variety of unnatural modifications [34]. These chemical modifications are often implemented in combination with structural features which improve the therapeutic activity of the peptide in order to maximize its clinical potential. Cyclic peptides, for example, often poses more favourable protein-binding properties compared to linear peptides [35]. Although many therapeutic challenges can be overcome through careful implementation of a variety of structural features, designing novel peptide sequences which fulfil all of the above criteria without compromising their therapeutic potential can be extremely challenging [36].

**Figure 2:**
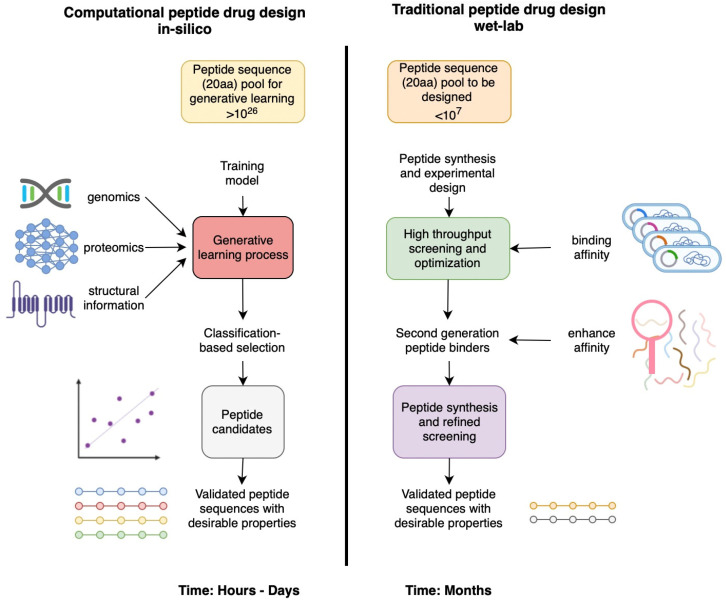
Comparison of a computational in silico pipeline vs. traditional high throughput wet-lab approach for designing therapeutic peptides: steps and timelines.

Phage display has quickly become a primary method to discover novel binding peptides that may have therapeutic potential [37]. Phage display is a powerful technique used in the discovery and development of peptide drugs. It involves the use of bacteriophages, which are viruses that infect bacteria, to display peptide libraries on their surface [38]. By genetically engineering the bacteriophage genome, researchers can link specific peptides to the coat proteins of the phage particles. Phage display offers several advantages in the discovery of peptide drugs. Firstly, it allows for the screening of a high number of peptide sequences simultaneously, providing a more extensive exploration of the peptide sequence space. Secondly, it enables the selection of peptides that specifically bind to a desired target, facilitating the identification of lead candidates for further development. Phage display can also allow for the identification of peptides with various properties, such as binding affinity, specificity, and functional activity [39]. While phage display is a widely used technique for identifying peptide drugs, it also has certain limitations. For example, although phage-display libraries can be large and diverse, the number of peptides that can be effectively screened is still limited compared to the vast AI-generated chemical space of peptide sequences. For example, a phage library that can encode 10^7^ peptides can only, optimistically, search 0.00000000000001% of the sequence space that is available to a 20-amino-acid peptide. This limitation may obviously result in missing potential lead peptide candidates with desired properties not represented in the library. Secondly, peptides displayed on the surface of phage particles may adopt different conformations than they would in their natural, biologically relevant state [40]. This conformational constraint may affect their binding properties, potentially leading to false positives or false negatives in the selection process. The phage-surface peptides are also differentially expressed, prone to incomplete post-translational modifications and with a multivalent display that poses a higher risk of selection of less-specific binders due to the avidity [41]. In contrast, AI offers a transformative approach to peptide drug discovery by leveraging computational power, data analysis, and machine learning algorithms. AI algorithms can analyze vast amounts of data from various sources, including protein databases, genomic data, and chemical libraries, to identify patterns, relationships, and predictive models that can accurately depict peptide biological and physiochemical properties. This has the potential to enable researchers to make more informed decisions in peptide design, screening, and optimization for drug discovery applications (Figure 2) [14,42].

AI-driven peptide design involves the use of algorithms that can generate and evaluate large numbers of peptide sequences based on desired properties, such as target affinity, selectivity, and bioavailability. These algorithms can explore large chemical spaces more comprehensively and efficiently than traditional methods. By integrating structural and functional data, AI models can precisely predict peptide–protein interactions and accurately guide the rational design of peptides with improved binding capabilities. Moreover, AI-based screening approaches can accelerate the identification of lead candidates by analyzing high-throughput screening data and learning from the patterns and properties of active molecules. Machine learning models can also predict peptide bioactivity and toxicity [43], enabling the prioritization of promising candidates and reducing the need for extensive experimental testing.

While traditional approaches rely heavily on experimental validation, AI offers a data-driven approach that complements and enhances experimental quality-control efforts. By combining the strengths of both experimental and computational approaches, AI can significantly improve the efficiency, success rates, and overall quality of peptide drug discovery [44]. For example, it is easy to imagine a drug discovery pipeline that implements an initial AI design platform of peptide candidates to prioritize and expedite downstream experimental validation efforts. However, the implementation of AI in peptide drug discovery is not without challenges. AI models heavily depend on the availability of high-quality and diverse training datasets [20]. Data limitations, including biased datasets or limited structural information, can impact the accuracy and generalizability of AI models. To compensate for this, score and rank functions, with statistical significance, provide an element of reliability and reproducibility in AI-generated results. Additionally, the interpretability and explainability of AI-driven predictions in peptide drug discovery remain important areas of research, as the decision-making process should be transparent and scientifically sound.

## 3. AI-Driven Peptide Design and Optimization

AI-driven approaches have already begun to transform the field of peptide design and optimization by leveraging computational algorithms and predictive models to guide the discovery of novel peptide therapeutics [45]. Collectively, these approaches enable researchers to explore a vast chemical space, predict peptide–protein interactions, and optimize peptide sequences for enhanced efficacy and specificity against therapeutic targets.

Rational peptide design using AI algorithms is a key component of AI-driven approaches. Machine learning models, such as support vector machines, random forests, or neural networks, can be trained and integrated in large datasets of known peptide–protein interactions to learn patterns and predict binding affinities. These models can then be used to design peptides with improved affinity and selectivity for specific targets. For example, the In Silico Peptide Synthesizer (InSiPS) is a genetic algorithm to create a massively parallel computational tool used to generate novel, synthetic protein sequences designed to interact with a specific target while avoiding off-target interactions [46]. InSiPS relies on the Protein–protein Interaction Prediction Engine (PIPE) to predict peptide–protein interactions (PPIs) which can overcome the limitations of experimental methodologies and is able to perform proteome-wide PPI predictions within a wide range of organisms with a low false positive rate (<0.05%) [17,46]. Algorithms, like InSiPS, can explore different peptide sequences by considering amino acid properties, physicochemical characteristics, and inherent structural features. By inputting desired target profiles, such as binding affinity or selectivity, into AI models, researchers may be able to generate peptide sequences that are optimized for specific therapeutic purposes. This rational design process reduces the need for extensive experimental screening and allows for the identification of promising candidates more efficiently. Moreover, AI-driven peptide design approaches can incorporate additional constraints and considerations, such as peptide stability, solubility, and immunogenicity. Machine learning models trained on large-scale datasets of peptide properties could predict and optimize these properties during the design process. This integration of multiple factors ensures that the designed peptides not only exhibit high target affinity, but also possess favorable drug-like characteristics [8].

Molecular modeling and docking tools also enable a structure-based design approach for building and refining peptide drugs [47]. Molecular docking functions as a computer-aided drug design platform based on available and reliable structural (X-ray or NMR) data of peptide ligand and protein targets, in free- and bound-complex form. Many programs have been developed to study and optimize the requisite peptide–protein binding interactions and structures that contribute to biological activity. For example, DOCK [48], and AutoDock [49] use a parameter set based on the AMBER force field [50] to define flexible and fixed ligand conformations with a target during the docking procedure. Molecular modeling and docking have also been implemented in the discovery of novel binding sites (and binding modes) of peptides and proteins without prior knowledge of structure and bound conformations. These studies have led to the prediction and optimization of energetically favored peptide–protein complex structures for refinement of bio-active peptides, including agonists, antagonists, antimicrobial, anticancer and immunomodulatory peptides [51]. Applications of molecular docking also extend to the investigation of protein–protein interactions [52].

Thus, AI plays a crucial role in predicting peptide–protein interactions. AI models can analyze protein structures, binding sites, and known interactions to elucidate the key features that contribute to peptide–protein binding [53]. By combining this structural information with machine learning algorithms, researchers can predict and evaluate the binding affinity and specificity of designed peptides for their target proteins. AI algorithms can also aid in the prediction of peptide secondary structures and conformational changes upon binding, which are crucial for understanding peptide–protein interactions [54]. This information is vital for optimizing peptide sequences to ensure proper folding and optimal binding to target proteins. However, to validate and refine AI-generated peptide designs, experimental validation is essential. For example, AI-generated results can be experimentally validated using mass spectrometry, nuclear magnetic resonance, crystallography and surface plasmon resonance, among other approaches, which validate peptide sequence, structure and binding activity against protein (receptor) targets. This iterative process of AI-driven design and experimental validation enables continuous improvement and refinement of the AI models, resulting in more accurate predictions and better designed peptide candidates.

In conclusion, AI-driven peptide design and optimization has the potential to revolutionize the discovery of novel peptide therapeutics. By leveraging computational algorithms, machine learning models, and predictive analytics, researchers can expedite the design process, accurately predict peptide–protein interactions, and optimize peptide sequences for enhanced efficacy and specificity against therapeutic targets [55]. The integration of multiple data sources and constraints allows for the generation of peptides with improved drug-like properties and a higher likelihood of success in therapeutic applications. AI-driven approaches complement experimental validation and offer a powerful toolset to revolutionize peptide drug discovery and facilitate the development of personalized and targeted therapeutics.

## 4. AI-Based Peptide Screening and Selection

AI-based peptide screening and selection approaches have significantly transformed the drug discovery process by accelerating the identification of lead candidates and streamlining the evaluation of peptide bioactivity and toxicity [55]. These computational methods leverage machine learning algorithms and predictive models to analyze large datasets and predict the properties and behavior of peptides, enabling researchers to prioritize promising candidates for further experimental validation. For example, RosettaScripts is a software framework developed by the Rosetta protein structure prediction and design suite [56]. It utilizes a combination of machine learning algorithms and physics-based modeling to predict and optimize peptide–protein interactions. RosettaScripts allows researchers to define custom protocols for peptide design and optimization by specifying the desired properties and constraints. The program can incorporate various scoring functions, energy models, and conformational sampling methods to assess and refine peptide–protein interactions [57]. Using a combination of machine learning and physics-based algorithms, RosettaScripts predicts binding affinity, specificity, and other relevant physicochemical properties of peptides for specific protein targets. It can guide the selection of peptide sequences with optimal binding characteristics, reducing the time and resources required for experimental screening. One notable application of RosettaScripts is its use in the design of peptide inhibitors for HIV-1 protease [58]. By combining machine learning algorithms with protein structure information, RosettaScripts has been able to predict and optimize peptide sequences that effectively bind to and inhibit the activity of HIV-1 protease, a critical enzyme for the replication of the HIV virus.

Machine learning algorithms, such as random forests, support vector machines, or deep-learning neural networks, are commonly employed to develop predictive models for peptide bioactivity. These models utilize a wide range of molecular descriptors, including physicochemical properties, structural features, and sequence patterns, to capture the complex relationship between peptide structure and biological activity [59,60,61,62]. By analyzing these descriptors, AI models can generate predictions regarding peptide potency, efficacy, and selectivity for specific targets. AI-driven peptide screening approaches also have the capability to optimize peptide properties beyond bioactivity and toxicity. These approaches can incorporate additional considerations, such as pharmacokinetic properties, drug-likeness, and synthetic feasibility to prioritize peptides with improved pharmacological profiles. By combining predictive models for pharmacokinetics, absorption, distribution, metabolism, and excretion (ADME), AI can guide the selection of peptides that possess favorable drug-like characteristics, increasing their likelihood of success in drug development [63].

As previously mentioned, to validate AI-generated predictions, experimental validation of selected peptides is crucial. Predicted peptide sequences can be readily synthesized and tested in various biological assays to assess their bioactivity, selectivity, and therapeutic potential. The experimental data obtained from validation studies can be used to refine and improve the AI models, enabling iterative optimization and enhancing the accuracy of future predictions. For example, InSiPS predicted peptides that bound to the SARS-CoV-2 spike (S) surface protein, to use as a diagnostic method for COVID-19. The results were experimentally validated using mass spectrometry and surface plasmon resonance approaches. Furthermore, the experimental data facilitated the discovery of additional SARS-CoV-2 peptides present in patients’ saliva, offering a potential avenue for the development of COVID-19 peptide diagnostics [64].

In conclusion, AI-based peptide screening and selection approaches provide a powerful means to prioritize and identify promising peptide candidates for drug discovery. By leveraging machine learning algorithms and predictive models, these approaches enable the analysis of large chemical libraries, the prediction of peptide bioactivity and toxicity, and the integration of multi-omics data for personalized medicine [14]. The incorporation of additional considerations, such as pharmacokinetic properties, further enhances the selection process, leading to the identification of peptides with improved drug-like characteristics [29]. AI-driven peptide screening and selection approaches streamline the drug discovery process, saving time and resources, and reducing the risk of failure in subsequent experimental validation.

## 5. Implementation

Several case studies and examples highlight the successful application of AI in the generation of new peptide drugs, showcasing its potential to revolutionize the field of peptide drug discovery. These examples demonstrate how AI-driven approaches have accelerated the identification of novel peptides with improved therapeutic properties, validated through experimental validation and clinical development. One notable example is the application of AI in the discovery of peptide-based inhibitors targeting specific protein–protein interactions. AI algorithms have been employed to analyze protein structures, identify key binding sites, and predict peptide sequences that can disrupt the targeted protein–protein interaction. By integrating structural information with machine learning models, researchers have successfully designed peptides with enhanced binding affinity and specificity for targeted protein–protein interaction [65]. Kozlovskii and Popov [66] introduced BiteNet(P)(p), a deep learning method utilizing 3D convolutional neural networks (CNNs) for protein–peptide binding-site detection. The model processes protein structures as 3D images, making it particularly suitable for large-scale analysis. In their approach, peptide-binding sites are represented as “hot spots” within the protein structure, identifying regions that contribute most to the protein–peptide interaction. The model was trained using a non-redundant dataset of protein–peptide complexes retrieved from the Protein Data Bank (PDB). The training was further enhanced by initializing the network with weights from a protein–ligand binding-site prediction model, enabling more accurate predictions for protein–peptide interactions. AI-designed peptides have shown promising results in preclinical studies, demonstrating their potential as therapeutic candidates for diseases such as cancer, autoimmune disorders, and infectious diseases [67,68].

Another case study showcases the use of AI-driven peptide screening in identifying peptide candidates with potent antimicrobial activity. Due to the high costs and the low profitability of developing new antibiotics, many large pharmaceutical companies have ceased their search for novel antibiotics. Additionally, the rapid emergence of microbial resistance has further discouraged these efforts. However, antimicrobial peptides have gained attention as promising alternatives, with a significantly lower rate of resistance development compared to traditional antibiotics. Traditional methods for discovering antimicrobial peptides rely on labor-intensive and expensive experimental screenings [69,70]. AI models trained on large datasets of antimicrobial peptides and their properties have accelerated the identification of peptide candidates with antimicrobial activity. By considering physicochemical properties, sequence patterns, and structural features, AI algorithms have successfully predicted the antimicrobial potential of untested peptides. The deep learning model AMPs-Net, trained on approximately 25,000 peptides sourced from 19 publicly available databases, has demonstrated superior performance compared to other models. It shows an improvement in average precision of 8.8–19.02% and in accuracy of 5.74–24.23%. As such, AMPs-Net demonstrates potential in predicting the antibacterial and antiviral capabilities of a wide range of antimicrobial peptides. Notably, the model successfully predicted the antimicrobial effects of two peptides, FE23 and KS22, which were subsequently validated in vitro using *E. coli* and *S. aureus*. [69]. Approaches like AMPs-Net have facilitated the discovery of novel antimicrobial peptides with broad-spectrum activity and a reduced likelihood of fostering microbial drug resistance.

AI has also been instrumental in the design and optimization of peptide therapeutics for targeted drug delivery. By analyzing protein expression profiles, genetic variations, and disease-specific factors, AI models have guided the development of personalized peptide therapeutics. This class of cell-targeting and penetrating peptides, and their related peptidomimetics, can selectively target diseased cells or tissues while minimizing off-target effects for selective drug-delivery applications [71]. For instance, AI-assisted peptide design has been used to develop targeted cancer therapies that selectively bind to cancer cells, enhancing treatment efficacy and reducing side effects associated with conventional chemotherapy [8]. One remarkable example of this is the development of an AI-based system by Tan et al. [72] which accurately predicts peptide half-life by incorporating enzymatic cleavage descriptors alongside traditional peptide descriptors and utilizing a transfer-learning strategy. Future advancements in AI-driven optimization of peptide modifications can result in the development of peptides with prolonged half-life, enhanced bioavailability, and improved therapeutic efficacy [73].

These case studies and examples demonstrate the transformative impact of AI in peptide drug discovery. AI-driven approaches have accelerated the identification of novel peptides, improved screening and selection processes, optimized peptide synthesis and modification strategies, and facilitated the development of personalized and targeted peptide therapeutics. As AI technologies continue to advance and datasets expand, the integration of AI in peptide-based drug discovery holds great promise for the development of more effective and precise peptide-based treatments for a wide range of diseases.

## 6. Future Prospectives and Challenges

The future prospectives of AI in the generation of new peptide drugs are promising, with the potential to further revolutionize the field of peptide drug discovery. However, several challenges need to be addressed to fully realize the potential of AI-driven approaches and ensure their successful integration into the drug development pipeline. One of the key requirements is based on the expansion and refinement of AI models through the incorporation of more diverse and comprehensive datasets. The availability of open-source, large-scale peptide databases, protein structures, omics data, and clinical data presents an opportunity to enhance the accuracy and predictive power of AI algorithms. By integrating multi-omics data, genetic variations, and patient-specific factors, AI models can facilitate the development of personalized peptide therapies tailored to individual patients or specific disease subtypes. Continued efforts to curate and annotate high-quality datasets will be essential in improving the performance and reliability of AI models in peptide drug discovery [74].

Another future prospective is the integration of AI-driven approaches with experimental screening techniques such as high-throughput and parallel library (micro) arrays and combinatorial chemistry [75]. By combining AI predictions with experimental validation in an iterative feedback loop, researchers can enhance the efficiency and success rate of peptide drug discovery [76]. This integration can facilitate the rapid identification and optimization of ‘*hit*’ peptide candidates, accelerating the translation of promising leads into clinical development. Furthermore, advancements in AI algorithms, such as deep learning and reinforcement learning, hold promise for improving the accuracy and interpretability of AI models. These algorithms can learn complex patterns and relationships in peptide data, leading to more accurate predictions and insights into peptide structure–activity relationships. Additionally, the development of explainable AI approaches will enable researchers to understand and interpret the decision-making process of AI models, enhancing trust and facilitating their adoption in the pharmaceutical industry [77].

Despite these prospectives, several challenges need to be addressed to fully harness the potential of AI in peptide drug discovery. One significant challenge is the availability of high-quality, annotated datasets for training AI models. Data collection, standardization, and curation of results remain critical to ensure the reliability and generalizability of AI predictions [78]. Efforts should be made to promote data sharing and collaboration among researchers and pharmaceutical companies to create comprehensive and diverse datasets that capture and advance the complexity of peptide–drug interactions. Another challenge is the interpretation and validation of AI-generated results [79]. While AI models can provide valuable predictions and insights, experimental validation is crucial to confirm the accuracy and relevance of these predictions. Developing robust experimental validation strategies and integrating them into the AI workflow will help validate and refine AI models, ensuring their effectiveness and reliability in guiding peptide drug discovery.

Ethical considerations and regulatory challenges are also important factors to address. As AI becomes more integrated into the drug development process, ensuring transparency, fairness, and accountability in AI-driven approaches is essential. Ethical guidelines and regulations should be established to address concerns such as data privacy, bias, and the responsible use of AI technologies in healthcare [80]. Collaboration among researchers, regulatory bodies, and industry stakeholders will be crucial in developing guidelines that promote the ethical and responsible use of AI in peptide drug discovery [81]. With that being said, the AI-generated peptide space is relatively new and rapidly evolving. As of today, there are no AI-generated peptide therapeutics commercially available on the market. However, many innovative peptide therapeutics are in various early stages of development, including preclinical studies with a few advancing to early-phase clinical trials, as of late 2024.

In conclusion, the future prospectives of AI-driven research in the generation of new peptide drugs are limitless. Advancements in AI algorithms, the integration of diverse datasets, and the collaboration between academia, industry, non-profit and regulatory bodies can accelerate the AI-driven process towards the discovery of peptide therapeutics. Overcoming challenges related to data availability, validation, ethics, and infrastructure will be crucial for the successful implementation of AI in peptide drug discovery. With continued advancements and collaborative efforts, AI has the potential to transform the field, enabling the development of safer, more effective, and personalized peptide-based therapeutics for a wide range of diseases.

## Data Availability

Not applicable.
